# Drivers and rates of stock assessments in the United States

**DOI:** 10.1371/journal.pone.0196483

**Published:** 2018-05-11

**Authors:** Philipp Neubauer, James T. Thorson, Michael C. Melnychuk, Richard Methot, Kristan Blackhart

**Affiliations:** 1 Dragonfly Data Science, Wellington, New Zealand; 2 NOAA Northwest Fisheries Science Center, Seattle, WA, United States of America; 3 School of Aquatic and Fisheries Science, University of Washington, Seattle, WA, United States of America; 4 ECS Federal, INC., Fairfax, VA, United States of America, on behalf of NOAA Fisheries, Office of Science and Technology; Technical University of Denmark, DENMARK

## Abstract

Fisheries management is most effective when based on scientific estimates of sustainable fishing rates. While some simple approaches allow estimation of harvest limits, more data-intensive stock assessments are generally required to evaluate the stock’s biomass and fishing rates relative to sustainable levels. Here we evaluate how stock characteristics relate to the rate of new assessments in the United States. Using a statistical model based on time-to-event analysis and 569 coastal marine fish and invertebrate stocks landed in commercial fisheries, we quantify the impact of region, habitat, life-history, and economic factors on the annual probability of being assessed. Although the majority of landings come from assessed stocks in all regions, less than half of the regionally-landed species currently have been assessed. As expected, our time-to-event model identified landed tonnage and ex-vessel price as the dominant factors determining increased rates of new assessments. However, we also found that after controlling for landings and price, there has been a consistent bias towards assessing larger-bodied species. A number of vulnerable groups such as rockfishes (Scorpaeniformes) and groundsharks (Carcharhiniformes) have a relatively high annual probability of being assessed after controlling for their relatively small tonnage and low price. Due to relatively low landed tonnage and price of species that are currently unassessed, our model suggests that the number of assessed stocks will increase more slowly in future decades.

## Introduction

Fisheries scientists have measured human impacts on populations of finfishes and invertebrates for over 100 years with the goal of balancing the value derived from fishing with the long-term sustainability of populations [1]. This is principally achieved by estimating two measures of human impact: (1) fishing rate, i.e., the instantaneous mortality or annual fraction of the population that is harvested relative to an estimated target level, and (2) the population abundance, i.e., spawning biomass or reproductive output relative to an estimated target level. Together these measures reflect the “stock status” of an assessed population, and fisheries agencies are increasingly committed to maintaining fished populations at fishing rates below and population abundances above limit levels that are defined based on biological and economic considerations [2]. Where these quantities are most completely measured, global studies find that the most effective management occurs [3, 4].

The National Oceanic and Atmospheric Administrations’ National Marine Fisheries Service (NMFS, the agency in charge of federal fisheries management in the United States (US)) is committed to “end overfishing” for all marine species within regional fisheries management plans (with exceptions granted in certain circumstances; [2]). In the US, overfishing is defined as any stock having annual harvest rate or quantity above limit levels, generally set at the level that would produce maximum sustainable yield. A limit (or target) harvest quantity can in theory be calculated by combining a limit (target) harvest rate with an estimate of current population abundance. However, the vast majority of overfishing limits are currently estimated using methods that do not individually estimate either harvest rate or population abundance [5, 6]. For example, catch-only methods (COMs), such as depletion-corrected average catch (DCAC; [7]), are used to estimate an annual fishing limit for many data-limited stocks, but are not capable of estimating population abundance. COMs can therefore be used to help “end overfishing,” but are not otherwise informative about the status of a fished population.

Conservationists and ecologists will often be more interested in estimating population abundance (or abundance relative to equilibrium conditions) than estimating an overfishing limit [8]. Estimating abundance generally requires applying a population model to available harvest data and an index of population depletion (either an index proportional to population abundance, or average body size or age data). Estimated abundance is then compared to a biological reference point, or benchmark, for assessing current status relative to target levels. In the following, we consider this pairing of model-estimated abundance with estimated reference points as “stock assessments,” although we acknowledge that other authors have used the term “stock assessment” more broadly to also include methods that estimate overfishing limits but not population abundance. Although NMFS has estimated overfishing limits for the vast majority of fishes in US fisheries management plans, a smaller percentage of fished species has a stock assessment under our more restrictive definition. The dearth of stock assessments arises because developing a stock assessment typically requires more input data and time, and therefore requires extensive financial resources [9].

Stock assessments are important for many applied and theoretical questions regarding marine ecosystems. In particular, managing and monitoring rebuilding of overfished stocks to a target biomass level, rather than simply managing annual fishery removals, is possible only by estimating population abundance relative to target levels using stock assessment models. However, there is little previous research regarding which fished species are more or less likely to receive sufficient attention to develop a stock assessment. Understanding which species are more or less likely to be assessed could be useful for the following three reasons:

1. Conservation concerns and, conversely, missed opportunities for increased exploitation will be undocumented, so will receive less attention from the public and fishery managers.

2. Output from stock assessments has often been used in meta-analyses to understand ecological characteristics of marine fishes in general [10–12]. Therefore, it is important to understand the nature of systematic biases towards particular stock characteristics, as these will also bias our ecological understanding of marine fishes.

3. Stock assessments often require periodic updates (e.g., Pacific hake has been re-assessed annually from 1982 through 2016; [13]), and agency resources might be fully expended while assessing a small fraction of possible stocks. If the rate of assessing new species is decreasing, this could indicate the need for additional public resources for stock assessment or improved strategies for prioritizing which stocks to assess [14].

In this paper, we provide a quantitative analysis of which marine species landed by commercial fisheries are likely to have undergone a stock assessment using a statistical population dynamics model coupled with estimated reference points. We combine two databases representing fished coastal marine species in the continental US and Alaska: a database of landed tonnage and value by species from 1950 to 2013, and a database of management and stock assessment attributes for US fishes and invertebrates drawn from peer-reviewed stock assessments. We record the year that each stock with commercial or recreational catches in the continental US and Alaska (whether caught in US federal or state jurisdictions) first had a stock assessment, and we treat any stock that did not have an assessment by 2013 as a “censored” observation (i.e., it might eventually have an assessment). We then apply a censored time-to-event model to answer the following questions: (1) What economic and biological characteristics are associated with a high or low annual probability of being assessed for the first time?; (2) how has the rate of assessing stocks differed among four US regions (Northeast, Southeast, Alaska, and US West Coast) and with federal vs non-federal management authority?; (3) are there certain taxa (e.g., invertebrates, sharks, flatfishes, etc.) that are assessed substantially faster or slower after accounting for biological and economic attributes?; and (4) is the rate of stock assessment accelerating or decelerating over time? We show that landed tonnage and ex-vessel price are the main drivers of increasing rates of stock assessments, but larger fish and some taxa of conservation concern defy these trends and are more or less likely to be assessed.

## Methods

### Operational definition of US stock assessments

Many types of stock assessments are applied in the US, with varying levels of model complexity and input data requirements. Assessments for any given stock also tend to change over time, typically becoming more complex as warranted by available data. For consistency across US regions, we defined a stock assessment in this study as:

(A) a single-species model of density-dependent population dynamics (e.g., including some combination of individual growth, recruitment, or aggregate surplus production); where

(B) model parameters were estimated by fitting to abundance index and/or age or length compositional data;

(C) the model provided time series estimates of population abundance (e.g. total biomass, spawning biomass) and/or exploitation rates (e.g., fishing mortality or harvest fractions); and

(D) management benchmarks corresponding to these time series estimates were estimated within the assessment or were otherwise explicitly stated, where benchmarks included target reference points, reference points based on maximum sustainable yield (MSY) or its proxies, or initial population abundance; ratios of the time series and their corresponding reference points provide a relative index of stock status.

Age-structured models, delay-difference models, biomass dynamics models, and surplus production models all qualified as assessment models [15] as long as they also explicitly reported management benchmarks. We recognize that COMs such as stock-reduction analyses (SRAs) are often used to estimate overfishing limits for stocks in the absence of a population-dynamics model fitted to data [7, 16]. However, stock-reduction analyses did not qualify as stock assessments under this definition because they typically are not fitted to abundance-index or compositional data.

### Defining the set of landed stocks

The set of stocks for this analysis included all landed species of fish and invertebrates in US marine waters, and is not restricted to stocks listed in federal fishery management plans (FMPs; stocks that NFMS has jurisdiction over). We used stock units defined for assessment purposes for all stocks for which assessments were available. In most cases, these will be units that management decisions are made on.

We excluded some species based on practical considerations. Highly migratory species often have population boundaries that substantially exceed the jurisdiction of any single nation, and also are often difficult to assign to any one of the regions that we define for later analysis. We therefore excluded species that are typically assessed by Regional Fisheries Management Organizations, including tuna, billfish, and oceanic sharks (noting that some are managed by both RMFOs and federal FMPs). We also excluded salmon and shad from our analysis because assessments for these anadromous species are often conducted at a fine spatial resolution which might otherwise either numerically dominate the other landed marine species or conflict with the typical spatial resolution for marine stock assessments. We include mollusks, crustaceans and echinoderms, but exclude corals, sponges, and other benthic invertebrates as the latter have limited use as seafood. Finally, we exclude stocks landed or assessed in the US Pacific Islands and the Caribbean, which have not yet been added to our stock assessment database.

After excluding the above species, we used the US National Oceanic and Atmospheric Administration (NOAA) landings database [17] to identify landed stocks. This database provides annual landings for both assessed and unassessed species by state from 1950-2013. We aggregated state landings into four bio-geographic regions, defined as: Alaska (i.e., the Eastern Bering Sea, Gulf of Alaska, and Aleutian Islands); US West Coast (i.e., the marine waters of Oregon, Washington, and California); Northeast Coast (including the mid-Atlantic Coast); and Southeast Coast (including the South Atlantic Coast and Gulf of Mexico). Assignments of states to regions were generally unambiguous, except for differentiating stocks in Northeast and Southeast regions. For assigning state landings into Northeast vs. Southeast regions, we generally treated all states north of North Carolina (i.e., north of Cape Hatteras) as the Northeast region, and all other east coast states as the Southeast region. However, we made exceptions as several assessed stocks on the US east coast straddle our regional boundary; we assigned the assessed stock and its associated state landings to the region with the greatest average landings.

Landings of each species in each state were either assigned to an assessed stock or used to define an unassessed stock. For assessed stocks, we used areas of distribution as defined in assessments to determine which states’ landings were associated with that assessed stock. We considered occasional low-volume landings in nearby states to also belong to an assessed stock, because straying occurs and because fleets may catch fish in waters within a stock’s area of distribution but land fish in nearby states outside of that distribution. For example, the area of distribution for the South Atlantic/Gulf of Mexico finetooth shark stock comprises waters from North Carolina southward, but occasional landings in Virginia and Maryland were considered to also pertain to the assessed stock. However, if an assessment indicated that landings from only certain state(s) were considered for the assessment, we did not link landings in other nearby states to the assessed stock. Landings from states that were not linked to assessed stocks allowed us to define unassessed stocks. These state landings were pooled within each region to define the unassessed stock; thus, a maximum of one unassessed stock per region was defined for each species. For unassessed stocks, we therefore used a species-by-region definition of a stock, which amounts to the largest common denominator. For the most part, our region definitions correspond to bio-geographical regions, and few stocks straddle these boundaries (we exclude highly migratory stocks for this reason). Conversely, it may be that actual biological populations may be smaller than our regional definition and that corresponding assessments, were they to take place, would span only sub-areas. However, there is no basis for making such a decision *a priori*, and we decided on what appeared to be the most parsimonious stock definition for unassessed species.

Landings that were not resolved to species but only to higher taxonomic levels (e.g., “Scallops”) were excluded from our analysis. However, for some assessed stocks that were landed only as stock complexes prior to their first assessment, we inspected assessment documents for species-specific landings prior to the year of first assessment, and manually added these stocks to our final dataset to have as complete a record of assessed stocks as possible. As the NOAA landings database does not differentiate between wild-caught and farmed (aquaculture) landings, we obtained information about aquaculture landings from the NOAA Fisheries of the US report [18]. Most aquaculture landings over the past five years were from salmon, oysters, clams and mussels. To test the sensitivity of our results to the the inclusion of landings from the latter three species groups (salmon are already excluded), we repeated all analyses without these groups (i.e., by discarding a total of 21 stocks from the landings database). As the results were nearly identical to those found when including these stocks (results not shown), we chose to maintain these stocks within our dataset.

### Defining year of first stock assessment

Given the set of assessed stocks in each of the four US regions, we determined the year of first assessment for each of them. Identifying assessed stocks and their year of first assessment was accomplished by a combination of interviews with regional stock assessment scientists and literature reviews of archived assessments (S1 Appendix). For quality control, we compared our assignments of first assessment year with the NOAA Species Information System (SIS) database to ensure consistency for federally managed species (S2 Appendix). The SIS database does not contain information about when a stock was first assessed for the entire period considered here, but does contain information about the first assessment for some recently assessed stocks. Therefore, comparisons were restricted to the most recent SIS classification. These comparisons generally showed consistency among datasets, with categories of “Levels of Stock Assessment Models” in SIS aligning with our assignments of first stock-assessment year (as defined by criteria A-D above). Of the 211 stocks for which we assigned a year of first assessment, there were 28 discrepancies with SIS classifications which resulted from violation of criteria A-D (see S2 Appendix). These stocks were previously assessed using population models, but are either currently assessed with less complex methods or have had the most recent assessment rejected. For our analyses, we continue to consider these stocks as “assessed” and use the year that they were first assessed by a population dynamics model as their year of first stock assessment.

### Explanatory variables

Several variables were considered as explanatory factors affecting the year in which a stock was first assessed. Region and habitat typically occupied by the population were each treated as categorical random effects. Habitat types from FishBase [19] or SeaLifeBase [20] were compiled in R using rfishbase [21] and aggregated into six categories: deep sea (>200m; bathy-pelagic or bathy-demersal); benthic; demersal; benthopelagic; pelagic; and reef-associated. Maximum body length of the species was also assigned to each population and used as a numerical predictor, drawing from FishBase and SeaLifeBase. The catch quantity and ex-vessel price of the population together determine landed value of the population; more valuable populations may be more likely to be assessed. We considered maximum annual landings prior to the first assessment and mean ex-vessel price (US$ ⋅ kg^−1^) prior to the first assessment as separate numerical predictors, drawn from the NOAA landings database. Note that the database starts in 1950 and these values may therefore be biased for stocks for which maximum landings occurred prior to 1950 or had substantial landings from foreign fleets, or for which the mean ex-vessel price has changed substantially since 1950. However, given that we consider time-to-assessment from 1960 (see next section), we have at least 10 years of data for stocks landed prior to 1950. The full dataset is provided in the supplementary material (S1 File).

To evaluate whether the presence of particular stocks in a federal FMP changed the time-to-assessment, we performed our analysis both with and without this factor included as a binary fixed effect in our model detailed below. However, given the potential collinearity between this factor and other explanatory variables (i.e., the same factors that drive stock assessments may also drive inclusion in FMPs. While a stock assessment often precedes inclusion in a FMP, the reverse does occur for some stocks, and stocks can be placed in a FMP to facilitate data collection and stock assessment), we used our model without this factor to evaluate effects of other covariates.

### Time-to-event model

To assess which factors drive the overall rate of assessments and the time from first recorded landings to a full stock assessment, we applied a time-to-event model. These models account for censored data (i.e., species that are landed but not yet assessed) while modeling time-to-assessment within a parametric framework. The first stock assessment (as defined by our criteria above) occurred in 1960, and we therefore used 1960 as the first possible assessment year for stocks that were first landed prior to 1960. We thus assume, based on the first recorded assessment, that the necessary technology (models, computers to fit models, stock status reporting requirements, etc.) was not available prior to 1960 to conduct a full stock assessment as we have defined. Thus *T* = min(*Y*_*a*_ − *Y*_*l*_, *Y*_*a*_ − 1960), where *Y*_*a*_ is the year of first assessment for assessed stocks or 2013, the last year in our database, for unassessed stocks, and *Y*_*l*_ is the year of first landings in the NOAA database (starting in 1950). In other words, we defined time-to-event (*T*) as the time between 1960 (or first recorded landings if post-1960) and the first full stock assessment or, for unassessed stocks, the last record in our database (2013).

The Weibull distribution is often used as a flexible model that has several desirable properties for this type of analysis, and one can easily check whether the Weibull distribution is appropriate for the data at hand (see S1 Fig). The shape parameter of the Weibull density can be interpreted in terms of the rate of events occurring. A shape parameter >1 suggests an increasing rate of events, whereas a shape parameter <1 indicates a decreasing rate. This allows us to directly estimate the change in assessment rates over time.

A further desirable property is that the estimated regression coefficients can be interpreted both in terms of the ratio of event rates as well as time-to-event probabilities. For example, one can interpret a model coefficient as decreasing or increasing the likelihood of an event occurring at any particular time relative to the baseline (this is usually called the hazard ratio interpretation). Coefficients are estimated for explanatory variables and thus indicate the level and direction of influence of the variable on the base rate of assessment. A coefficient can also be transformed to allow a time-to-event interpretation, where time-to-event parameters represent a multiplicative increase or decrease in the expected time until an event occurs. For example, in a hypothetical scenario, the median time-to-assessment of a demersal stock may be 0.5 times that of a pelagic stock, suggesting that it takes twice as long for pelagic stocks to get assessed. Such acceleration factors are just transformations of the parameters obtained for the event rate interpretation—the two interpretations are easily exchangeable in the Weibull model.

We thus model time-to-assessment as Weibull-distributed with shape parameter *τ* and rate λ:

$$\begin{array}{c} T∼Weibull(\tau ,\lambda ) \end{array}$$ (1)

To convert from the estimated event rate to the time-to-event interpretation, we write the Weibull density as a function of the product of the rate *r*(*t*) at which assessments occur, and the probability *A*(*t*) of the assessment not occurring prior to time *t*.

$$\begin{array}{ccc} f(t) & = & A(t)\times r(t) \end{array}$$ (2)

$$\begin{array}{ccc} & = & \exp\left(-\lambda t^{\tau }\right)\times \lambda \tau t^{\tau -1}, \end{array}$$ (3)

where *A*(*t*) = 1 − *P*(*T* ≤ *t*) = 1 − *F*(*t*), with *F*(*t*) = exp(−λ*t*^*τ*^) the Weibull distribution function.

We modeled the scale λ of the Weibull distribution as a linear combination of covariates and categorical random effects via a log-link function:

$$\begin{array}{c} log\left(\lambda_{i,r,h,c,o,f}\right)=\beta X_{i}+\alpha_{r}+\gamma_{h}+\kappa_{c}+\omega_{o}+\zeta_{f}, \end{array}$$ (4)

where *β* is a row-vector of regression coefficients, and *X*_*i*_ is a vector of continuous covariates as well as the binary FMP (fixed) effect. Continuous covariates were taken as the (base 10) logarithms of mean ex-vessel price, maximum landings, their interaction (i.e., mean ex-vessel price × maximum landings) and species maximum length, all standardized by twice the standard deviation of the variable to allow comparison with the binary predictor for presence in FMPs [22]. Categorical variables other than presence in FMPs, *α* (region), *γ* (habitat), *κ* (class), *ω* (order), and *ζ*_*f*_ (family) were all treated as random effects. The model was fit within a Bayesian framework, using Markov Chain Monte Carlo (MCMC) as implemented in the JAGS package. MCMC was run using three chains of 210 000 iterations each, keeping every 100th iteration, with 10 000 iterations for each chain discarded as burn-in. This provided 6 000 samples from the posterior distribution for each parameter.

The variance of each random effect was given a half-Cauchy prior with a scale of Θ = 100, regression coefficients had vague normal priors with a precision of 1/*σ*^2^ = 1*e*^−5^, and *τ* was estimated using a gamma distribution prior with parameters *a* = *b* = 1*e*^−5^.

## Results

**Fig 1:**
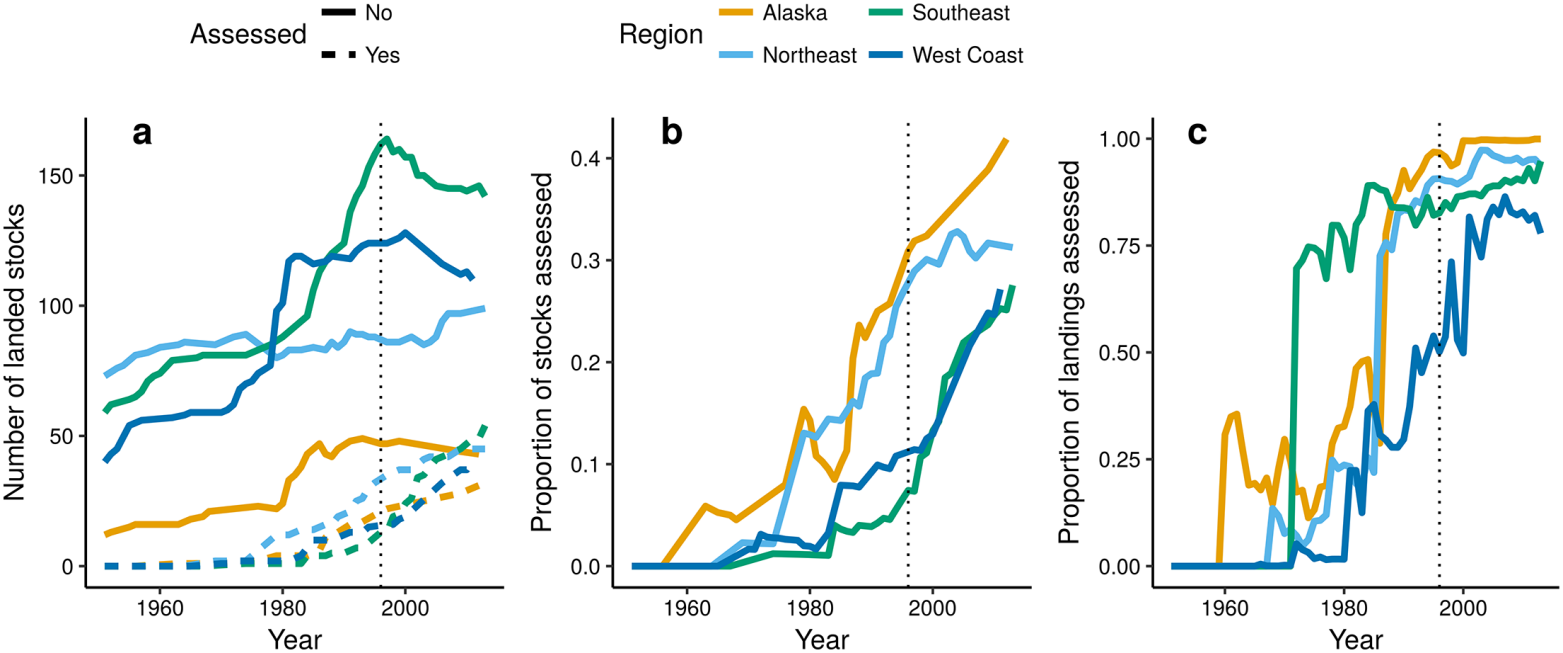
Time-line of a) the number of stocks landed by region and assessment status, b) proportion of landed stocks that are assessed, and c) the proportion of landed tonnage derived from assessed stocks. Values in (a) and (b) are based solely on NOAA landings data resolved to species level, and exclude landings of higher taxonomic groupings (e.g., stock complexes). Some assessed stocks appear only as stock complexes prior to their first assessment; we manually added these stocks to our final dataset, but they lack a complete time-series of species-specific landings so do not appear in (a) or (b). The dotted vertical line marks the re-authorization of the Sustainable Fisheries Act in 1996 that required rebuilding of overfished stocks, and required biomass limits to be estimated.

The number of landed marine stocks in the US (excluding salmon, shad, some benthic invertebrate, and highly migratory species) increased steadily from the 1960s into the 1990s (Fig 1a). During this period, the number of landed stocks in Alaska, West Coast, and Southeast regions approximately doubled, while the number of landed stocks in the Northeast increased more slowly (but was already relatively high at the start of this period). Most of the newly-landed stocks were unassessed throughout this period; by 1996 (the year of the re-authorization of the Sustainable Fisheries Act that required rebuilding of overfished stocks, thus required biomass limits to be estimated), fewer than 30 stocks in each of the four regions were assessed according to the definition of assessment used here. As a proportion of all landed stocks, however, the trend in assessed stocks has steadily increased in all regions since the 1970s or 1980s (Fig 1b). Currently, the proportion of landed stocks in our dataset that are assessed ranges from 32% of 193 stocks landed in the Southeast to 51% of 83 stocks landed in Alaska. In terms of regional landings (using only landings that are resolved to species level), the assessment of stocks with high landings in each region between the 1970s and 2000s lead to rapidly increasing proportions of total landed tonnage being comprised of assessed populations. By 1996, >93% of landings in Alaska, Northeast, and Southeast regions were comprised of assessed stocks, and in the West Coast this proportion has increased rapidly from 50% in 1996 to >77% currently (Fig 1c).

**Fig 2:**
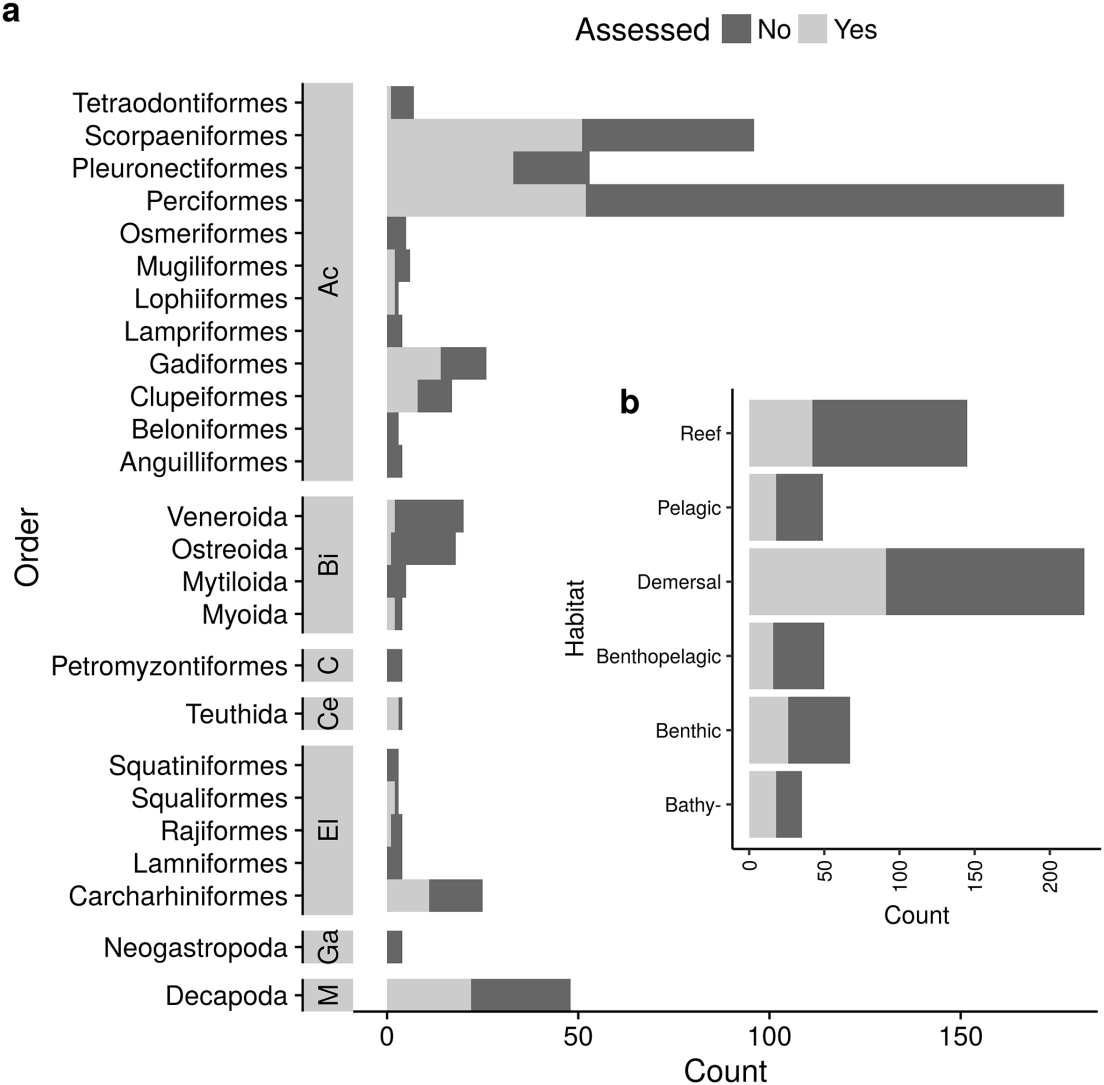
Assessment status at time of last known status (censoring time) a) by taxonomic order and sorted by class and b) by habitat type. In a), classes are abbreviated as Ac: Actinopterygii, M: Malacostraca, Bi: Bivalvia, El: Elasmobranchii, C: Cephalaspidomorphi, Ga: Gastropoda, and Ce: Cephalopoda. Only orders with more than three stocks are shown.

The majority of landed stocks were fish species (Fig 2a), with Perciformes, Pleuronectiformes and Scorpaeniformes dominating both the number of assessed and unassessed stocks. Among invertebrate taxa, decapod (crab) species were the most commonly landed and also most commonly assessed. Demersal species represent a higher proportion of landed populations than species associated with other habitat types (Fig 2b), and also accounted for the highest number and proportion of stock assessments.

**Fig 3:**
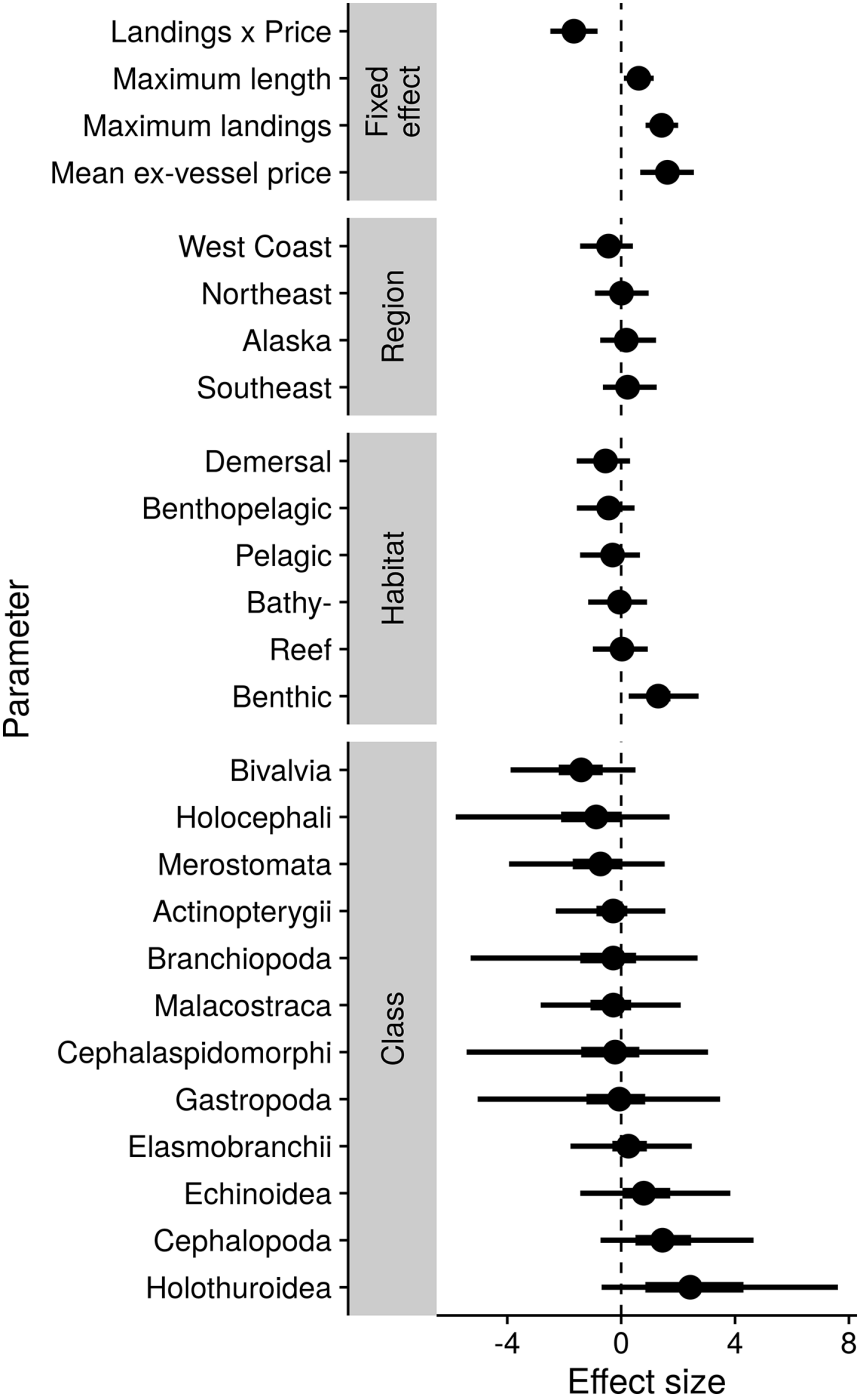
Summaries of estimated posterior distributions for fixed effects, regional random effects, habitat random effects, and taxonomic class random effects in the time-to-event model. Circles show posterior medians, thick bars show inter-quartile ranges of the posteriors, and thin lines show 95% confidence intervals. A positive effect size indicates an increased assessment rate (decreased time to first assessment).

Our time-to-event model effectively disentangled the biological and fishery characteristics that explaining differences in annual probability of first assessment among stocks (see S1 Fig and S2 Fig for model diagnostics). Among the numerical covariates considered (Fig 3, S1 Table), maximum annual landings and ex-vessel price both had positive and strongly significant impacts on annual assessment probabilities. The effect of landings on assessment probability therefore explains how each region has a large proportion of landed tonnage derived from assessed populations (Fig 1c), but a smaller proportion of landed stocks being assessed (Fig 1b). The interaction between price and landings was negative, suggesting that price is more influential when landings are small, and that the landed tonnage drives assessments for species with a low per-kg price (see also S3 Fig). The effect of maximum length was greater than zero, suggesting that large-bodied species have been preferentially assessed. However, the effect size (per two standard deviations) of maximum body length was smaller than that for price or landings.

Fifty-nine percent of 293 stocks in our analysis within federal FMPs had assessments meeting our criteria, compared with 13% of 276 stocks that were not in a FMP. In our time-to-event model, being within a FMP had more rapid assessments, but also lowered the effect size of other continuous covariates (S4 Fig), whereas an effect on categorical variables was not noticable.

**Fig 4:**
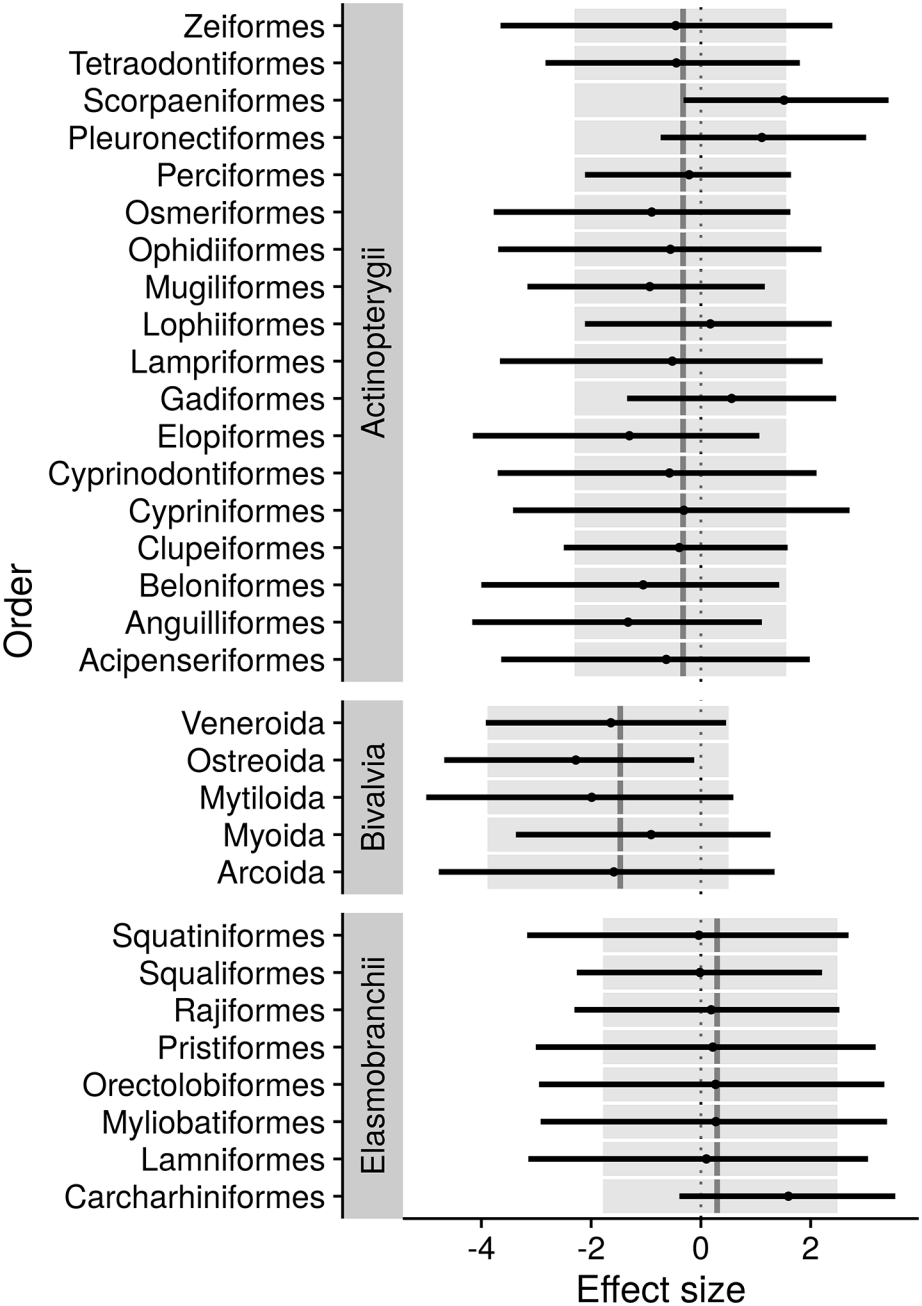
Summaries of estimated posterior distributions for random effects of orders within classes. For classes containing multiple nested orders in the dataset, grey lines show posterior means and coloured boxes show 95% confidence intervals of class effects. Order effects are shown as relative to the class effect within which they are nested, with points showing posterior means and black lines showing 95% confidence intervals.

Among explanatory random effects, taxonomic factors (order and class) explained a larger portion of residual variance than either habitat or region factors (S5 Fig). This is reflected in the probability of prior assessment in any given year after first being landed (Fig 3 and S6 Fig, S1 Table), for which sea cucumbers (Holothuroidea), squids (Cephalopoda) and sea urchins (Echinoidea) had a slightly higher probability of prior assessment than bony fishes (Actinopterygii) or other taxonomic classes. Rockfishes (Scorpaeniformes), groundsharks (Carcharhiniformes), and flatfishes (Pleuronectiformes) had the highest probabilities of prior assessment among taxonomic orders, each having a higher assessment probability relative to the average of their taxonomic classes (Fig 4, S1 Table). Gadids (Gadidae) also had a high assessment probability relative to the average for bony fishes, while oysters (Ostreoida) had a low probability, even relative to the already-low average for bivalves. Habitat and regional effects were generally smaller than taxonomic effects. After controlling for other factors, benthic species had a higher probability of assessment than species from other habitats.

**Fig 5:**
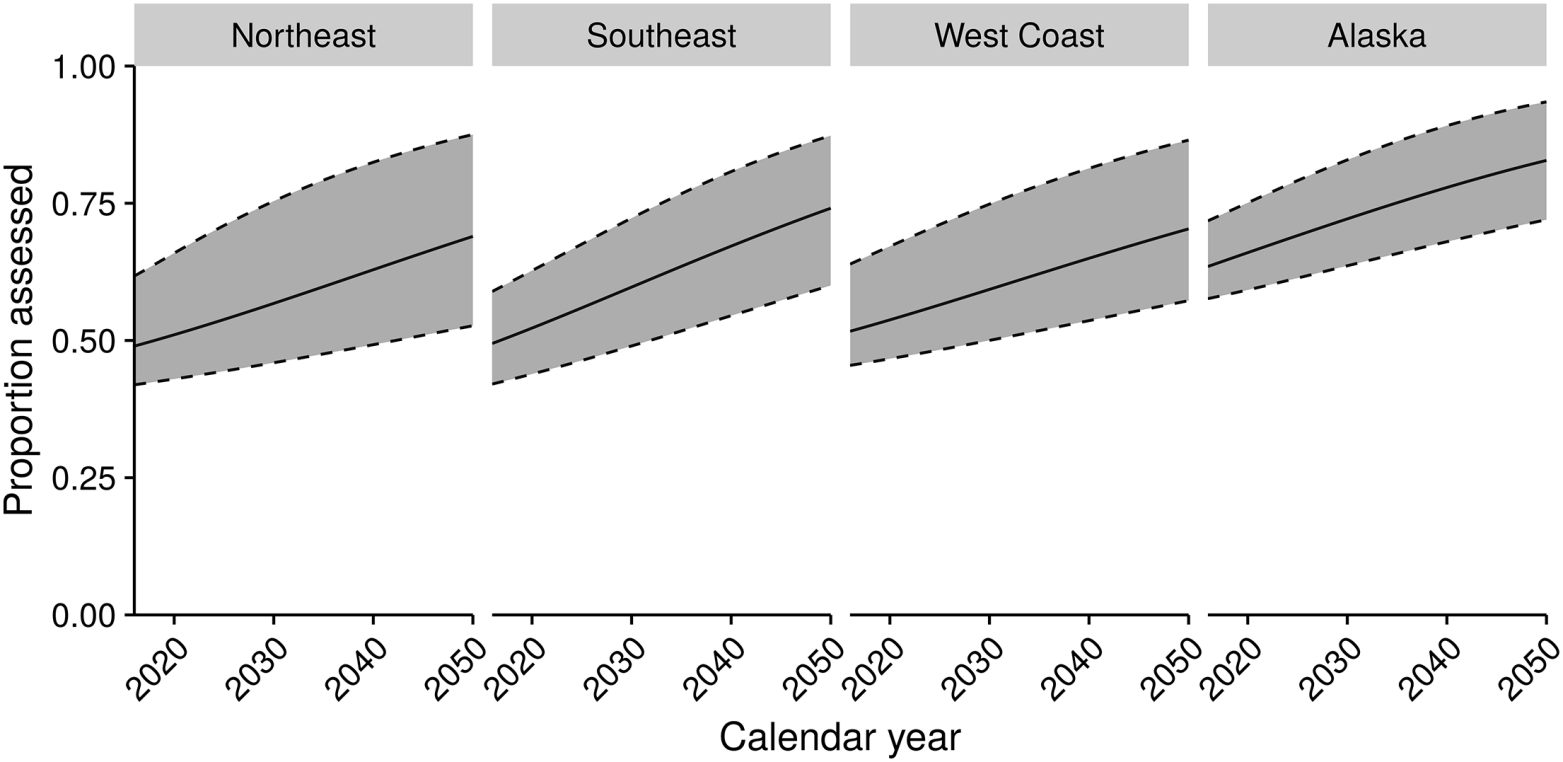
Projected proportion of stocks assessed by region and calendar year, based on assessment probabilities of stocks within each region over the projected year range.

Although our model suggests an increasing rate of assessments over time (posterior median for *τ*: 2.62), all regions show a relatively slow projected increase in the predicted proportion of assessed populations over the next three decades (Fig 5) compared to the rapid increases in the observed proportions of assessed populations over the last 35 years (Fig 2b). These projections rely on the values of maximum landings and ex-vessel price for all unassessed stocks, and were calculated from the final year of available time series data used for model fitting (usually 2013). The slow predicted increase occurs because stocks with a high assessment probability have typically been assessed, so that remaining stocks have low landings and prices, or other characteristics associated with low probability of assessment.

## Discussion

The status of assessed marine fisheries is generally found to be better than that of unassessed fisheries [23]; “what gets measured, gets managed” usually translates into improved sustainability of target stocks [3, 4]. NMFS currently estimates annual catch limits (ACLs) for the vast majority of fishes in US federal fisheries management plans, and has established accountability measures that are triggered whenever recorded annual harvest exceeds the ACLs [2]). Similarly, state-based management agencies also monitor catches and catch-per-unit-effort for many species, and implement management actions when this is deemed necessary. Thus, NMFS and other agencies both measure and manage annual harvest for the majority of US fishes. However, different methods are used for setting ACLs. Catch-only models (COMs) are used to estimate ACLs for the majority of stocks in most federal US management regions [5], and COMs do not estimate population size relative to management targets [16, 24]. In some cases, it may be possible to rebuild or maintain fish and invertebrate stocks at levels of sustainable harvest using COMs, without using a stock-assessment model as defined by our criteria [25, 26]. Specifically, COMs can be used to develop a harvest plan with fishing at a proportion of the estimated ACL, which is expected to have a pre-specified probability of maintaining population abundance near management targets [26]. Nevertheless, we have excluded COMs from our definition of “stock assessments”.

We see two main benefits to measuring population abundance for marine fishes beyond simply estimating ACLs:

1. COMs generally involve managing a fishery to target a constant annual harvest, which is chosen to perform adequately on average: some stocks may be overfished, others may be underfished, but the average fishing rate across all stocks is optimal. In addition, even optimal long-term target fishing removals may induce unsustainable fishing mortality if population abundance is low. In contrast, stock assessments are likely to perform adequately over time for each individual stock: some decades may be overfished, others may be underfished, but on average over time each individual stock is fished sustainably. Both approaches are expected to perform well on average, but stock assessments improve the expected performance for each stock individually. This advantage is important for both conservationists and fishers who do not wish to see any given individual stock overfished, irrespective of whether or not the average stock is overfished. Not only does overfishing pose a conservation challenge for depleted stocks, but may impose stricter fishing limits for other stocks as a result of bycatch limits for the depleted stocks [27, 28].

2. The ability of stock assessments to inform harvest plans based on updated data has been repeatedly shown to improve management outcomes [29]. For example, managing with a harvest control rule in which fishing mortality targets are updated based on stock assessment estimates of population abundance can substantially decrease variability in abundance and fishery catches (assuming the assessment does not change fundamentally over time), even relative to cases where a COM estimates sustainable fishing mortality rates perfectly [30]. Updating harvest plans based on new data can also prevent cases in which COMs over-estimate a sustainable fishing rate, which would otherwise collapse the fishery [26]. We therefore see benefits both to ocean conservation and to fishing industries by continuing to transition from management based on COMs to management based on stock assessments with associated biological reference points.

There are many differences in quality and complexity among stock assessments. NMFS categorizes assessments using six “levels”, and high-level assessments are distinguished by having more or higher-quality data assimilated, using a model that allows for greater attention to biological mechanisms and realism. We have ignored these subtler distinctions here, and have instead used a single cutoff criterion, which essentially falls between statistical population models and COMs. In general, our classification of unassessed stocks aligned with SIS categories 0–2, while our assessed stocks aligned with SIS categories 3–5 (S2 Appendix). We suggest that future research could build on our model to include annual probabilities of transitioning among multiple categories (e.g., among all six NMFS assessment levels). This future analysis would allow greater detail regarding historical changes over time in the average quality of stock assessments, and may show alternative patterns among regions dependent on the level of assessment complexity considered. NMFS has already embarked upon the task of defining and compiling records of different assessment types and qualities, so this research will soon be feasible for stocks in US-federal jurisdiction.

Given our operational definition of stock assessment, maximum landings and ex-vessel price were particularly strong predictors of the year in which stocks were first assessed. The product of landings and price is a rough measure of the gross economic value to commercial fisheries derived from fish and invertebrate stocks. Fisheries managers and scientists must choose among several candidate species in a given region and devote stock assessment time and resources towards only a subset of these. Our results suggest that fisheries managers prioritize stocks with high commercial value, and this result is consistent with previous research showing that fishery development is also driven primarily by landed tonnage and ex-vessel prices of fished species [31]. We were unable to identify a variable proportional to recreational value that was consistently measured across all regions, so we cannot estimate the potential impact of economic value for recreational fishing on assessment probabilities. However, we acknowledge that recreational landings and value will likely have an impact on both population dynamics and management in some regions, and especially for state-managed nearshore stocks.

A stock being listed in a federal FMP lead to accelerated time-to-assessment, while effects associated with landings and price were smaller when presence in an FMP was included in our analysis. This trade-off is likely driven by the strong dependence of presence in FMPs on landings and price. Indeed, logistic regression of the presence in a FMP against the same linear predictors used in our time-to-event model (without the FMP factor) shows similarly strong effects of landings and price, and qualitatively similar results to our time-to-event analysis (not shown). It thus appears that landings and price are primary drivers on the path towards assessment, and that placing stocks within FMPs increases the momentum towards a full assessment for these stocks.

We suspect that the positive effect of body size on time-to-assessment (after controlling for fishery value and landings) may be due to the large number and diversity of small-bodies stocks, especially invertebrates, that are landed in low numbers and at a relatively low ex-vessel price. Since these stocks do not have a consistent price or landings, and are taxonomically diverse, it may be more parsimonious within the model to attribute the lack of an assessment for these stocks to body length as opposed to taxonomic random effects.

Certain taxonomic classes, or orders within classes, stood out as being more likely to have undergone a stock assessment after controlling for landings, ex-vessel price, and other factors. Elasmobranchs, and in particular groundsharks (Carchariniformes), had relatively high rates of stock assessment when controlling for other variables. This likely results from increasing conservation interest in recent decades for shark species both in the US and worldwide [32]. This high assessment rate after accounting for maximum landings may also result in part from the high discard rates of small coastal shark species often caught as bycatch in shrimp trawl or other fisheries [33]. Due to bycatch, our database values for shark landings may be smaller than true harvest, thus resulting in a compensatory increase in the estimated assessment rate for this taxon. A similar effect may drive time-to-assessment probabilities of popular recreational species, for which the true harvest mortality is probably substantially higher than those reported in the NOAA landings database. Such under-reported total landings may explain higher-than-average assessment probabilities found for flatfishes (Pleuronectiformes), which are often estuarine or nearshore species. Among bony fishes, rockfishes and greenlings (Scorpaeniformes) also had high rates of assessment, likely due to the number of Pacific rockfishes included, which have been a topic of conservation concern in Alaska and the US West Coast [10, 34, 35]. While cephalopod abundance is commonly estimated using catch-per-unit-effort indices or survey abundance indices [36] rather than stock assessments, in the US most landed cephalopods are assessed (all are squid species). This may result from defined units of assessment having coast-wide distributions rather than assuming a more disaggregated stock structure in which only some of the stocks would be assessed.

Results from our model could be used to evaluate and control for systematic differences between assessed and unassessed US stocks in other analyses. These differences are important because meta-analysis of assessed stocks is widely used to understand management performance and biological characteristics of marine fishes in general [11]. To account for systematic differences between assessed and unassessed stocks, authors could use our model within a “propensity score matching” or “propensity score weighting” framework [37, 38]. For example, pairwise comparisons (or matching) between assessed and unassessed stocks should involve stocks with similar likelihoods of being assessed. Similarly, calculated propensity scores can be used as predictor variables in regressions involving variables of interest to control for the non-random assessment probabilities among analyzed stocks. If analysts find systematic differences in management outcomes or biological characteristics between assessed and unassessed stocks (e.g., systematic differences in recruitment compensation), then the relationship between the propensity of assessment and the variable of interest can be used to improve predictions for unassessed stocks.

Fish and invertebrate stocks in the US are reaching saturation with respect to the rate of first assessment. Even though most stocks in all regions are as yet unassessed (Fig 1b), the predicted rate of increase in assessed stocks over the next few decades is slower than the rate observed over the last few decades because the stocks most likely to be assessed have already been assessed. Furthermore, fisheries management agencies must weigh the benefit of attempting to obtain full assessments for new stocks against the need to update assessments of previously-assessed stocks. Since the latter have higher commercial value, and updating assessments is often more straightforward than collecting data and developing new assessments for previously-unassessed species, the percentage of assessed stocks is unlikely to attain 100%. Rather, at some point funding and resource limitations will likely lead to a steady state where no new stocks will be assessed and priority is given to updating existing assessments for stocks of high commercial, recreational and ecological value or concern. Nevertheless, priorities are subject to political and commercial pressures that are hard to predict, and our projections assume that these priorities do not change substantially from those in the past. Substantial changes in these influences could therefore lead to substantial deviations from our steady state projections.

It is not necessarily the case that stock assessments are required for effectively managing fish and invertebrate stocks, as harvest control rules or in-season adjustments to fishing effort can be based on fishery-independent survey indices or fishery-dependent catch-per-unit effort indices rather than on stock status estimates from assessments. However, a logical leap from “what gets measured, gets managed” to “what is better measured, is better managed” suggests the value of better estimating stock status through the use of stock assessments (population models fitted statistically to abundance index or age/length composition data, where estimated biomass is compared with explicit target levels). Further improvements in management performance given current resources could also be attained by improved methods (e.g., cost-effective methods to obtain trends for unassessed species [39, 40]) for prioritizing which stocks to assess, and we hope that the current results will help to inform ongoing plans to prioritize future stock assessments in the United States [14].

## Supporting information

S1 File Dataset used for analyses. The full dataset used for all analyses presented in the manuscript.
(XLSX)

S1 Table Paramter estimates. (PDF)

S1 Appendix Assignment of year of first stock assessment. (PDF)

S2 Appendix Validation of assessment classifications. (PDF)

S1 FigAppropriateness of the Weibull event-time model for the time-to-assessment dataset.If the Weibull applies, the time from first landings (or from first quantitative stock assessment in 1960 if a stock was landed before 1960) to the year of first assessment should fall on a line with slope *τ* (the Weibull shape parameter) between $log(-log\left(\hat{S}\left(t\right)\right))$, where $\hat{S}\left(t\right)$ is the non-parametric Kaplan-Meyer estimate of survival at time *t*, and the log of *t*. Here, *τ* evaluates to 1.71 (slope of the green line), suggesting an increasing assessment rate with increasing time *t*.(PDF)

S2 Fig Model fit of the Weibull survival model, based on Cox-Snell residuals calculated at the posterior mean of the linear predictor. For a perfect fit all data points would lie on the y = x (green) line.
(PDF)

S3 Fig Marginal probability of a stock being assessed after 50 years as a function of mean ex-vessel price (US.kg^-1^) and maximum landings prior to assessment. Marginal probabilities were evaluated at the mean of remaining continuous covariates. The dataset used for analysis is overlayed with assessed stocks as dark grey points and unassessed stocks in light grey.
(PDF)

S4 Fig Model estimates and predictions. Summaries of estimated posterior distributions for fixed effects, regional random effects, habitat random effects, and taxonomic class random effects in the time-to-event model with fishery management plan (FMP) variable. Circles show posterior medians, thick bars show inter-quartile ranges of the posteriors, and thin lines show 95% confidence intervals.
(PDF)

S5 Fig Comparison of finite population standard deviation (i.e., variance attributed to each variable) for random effects in the Weibull survival model. Circles show posterior medians, thick bars show inter-quartile ranges of the posteriors, and thin lines show 95% confidence intervals.
(PDF)

S6 Fig Marginal probability of a stock in category *k* being assessed as a function of time (*P*(*T*_*k*_ ≤ *t*) = *F*_*k*_(*t*) = exp(−λ_*k*_ *t*^*τ*^)), for stocks of various taxonomic orders, class, regions and habitats. For taxonomic variables, only the six levels with the most stocks represented in our dataset are shown. Marginal probabilities were evaluated at the mean of (centered) continuous covariates.
(PDF)

## References

[1] Smith TD. Scaling Fisheries: The Science of Measuring the Effects of Fishing, 1855-1955. 1st ed Cambridge, UK: Cambridge University Press; 2007.

[2] Methot RD, Tromble GR, Lambert DM, Greene KE. Implementing a science-based system for preventing overfishing and guiding sustainable fisheries in the United States. ICES Journal of Marine Science: Journal du Conseil. 2014;71(2):183–194. doi: 10.1093/icesjms/fst119

[3] Worm B, Hilborn R, Baum JK, Branch TA, Collie JS, Costello C, et al Rebuilding Global Fisheries;325(5940):578–585. 10.1126/science.1173146 19644114

[4] Melnychuk MC, Peterson E, Elliott M, Hilborn R. Fisheries management impacts on target species status. Proceedings of the National Academy of Sciences. 2017;114(1):178–183. doi: 10.1073/pnas.1609915114 10.1073/pnas.1609915114 PMC5224377 27994155

[5] Berkson J, Thorson JT. The determination of data-poor catch limits in the United States: is there a better way? ICES Journal of Marine Science: Journal du Conseil. 2015;72(1):237–242. doi: 10.1093/icesjms/fsu085

[6] Newman D, Berkson J, Suatoni L. Current methods for setting catch limits for data-limited fish stocks in the United States. Fisheries Research. 2015;164:86–93. doi: 10.1016/j.fishres.2014.10.018

[7] MacCall AD. Depletion-corrected average catch: a simple formula for estimating sustainable yields in data-poor situations. ICES Journal of Marine Science: Journal du Conseil. 2009;66(10):2267–2271. doi: 10.1093/icesjms/fsp209

[8] Hutchings JA, Minto C, Ricard D, Baum JK, Jensen OP. Trends in the abundance of marine fishes. Canadian Journal of Fisheries and Aquatic Sciences. 2010;67(8):1205–1210. doi: 10.1139/F10-081

[9] Dowling NA, Dichmont CM, Venables W, Smith ADM, Smith DC, Power D, et al From low- to high-value fisheries: Is it possible to quantify the trade-off between management cost, risk and catch? Marine Policy. 2013;40:41–52. doi: 10.1016/j.marpol.2012.12.009

[10] Myers RA, Bowen KG, Barrowman NJ. Maximum reproductive rate of fish at low population sizes. Canadian Journal of Fisheries and Aquatic Sciences. 1999;56(12):2404–2419. doi: 10.1139/f99-201

[11] Thorson JT, Cope JM, Kleisner KM, Samhouri JF, Shelton AO, Ward EJ. Giants’ shoulders 15 years later: lessons, challenges and guidelines in fisheries meta-analysis. Fish and Fisheries. 2015;16(2):342–361. doi: 10.1111/faf.12061

[12] Jensen OP, Branch TA, Hilborn R. Marine fisheries as ecological experiments. Theoretical Ecology. 2012;5(1):3–22. doi: 10.1007/s12080-011-0146-9

[13] Helser TE, Stewart I, Fleischer G, Martell S. Stock assessment of Pacific hake (whiting) in US and Canadian waters in 2006. Pacific Fishery Management Council. 2006;7700:97220–1384.

[14] Methot RD. Prioritizing fish stock assessments. Washington, D.C.: NMFS, NOAA, US Department of Commerce; 2015. Available from: https://www.st.nmfs.noaa.gov/Assets/stock/documents/PrioritizingFishStockAssessments_FinalWeb.pdf

[15] Dichmont CM, Deng RA, Punt AE, Brodziak J, Chang YJ, Cope JM, et al A review of stock assessment packages in the United States. Fisheries Research. 2016;183:447–460. doi: 10.1016/j.fishres.2016.07.001

[16] Dick EJ, MacCall AD. Depletion-Based Stock Reduction Analysis: A catch-based method for determining sustainable yields for data-poor fish stocks. Fisheries Research. 2011;110(2):331–341. doi: 10.1016/j.fishres.2011.05.007

[17] NOAA Fisheries Statistics Division. Annual Commercial Landing Statistics; 2016. http://www.st.nmfs.noaa.gov/commercial-fisheries/commercial-landings/annual-landings/index

[18] NOAA Fisheries Statistics Division. Fisheries of the United States; 2015. http://www.st.nmfs.noaa.gov/Assets/commercial/fus/fus15/documents/03_%20Aquaculture2015.pdf

[19] Froese R, Pauly D, et al.. FishBase; 2012. World Wide Web Electronic Publication, www.fishbase.org, version (10/2016).

[20] Palomares M, Pauly D. SeaLifeBase. 2010;.

[21] Boettiger C, Temple Lang D, Wainwright P. rfishbase: exploring, manipulating and visualizing FishBase data from R. Journal of Fish Biology. 2012;. doi: 10.1111/j.1095-8649.2012.03464.x 23130696 10.1111/j.1095-8649.2012.03464.x

[22] Gelman A, Hill J. Data analysis using regression and multilevel/hierarchical models. Cambridge university press; 2006.

[23] Costello C, Ovando D, Hilborn R, Gaines SD, Deschenes O, Lester SE. Status and solutions for the worldâ??s unassessed fisheries. Science. 2012;338(6106):517–520. doi: 10.1126/science.1223389 23019613 10.1126/science.1223389

[24] Wiedenmann J, Wilberg MJ, Miller TJ. An Evaluation of Harvest Control Rules for Data-Poor Fisheries. North American Journal of Fisheries Management. 2013;33(4):845–860. doi: 10.1080/02755947.2013.811128

[25] Wetzel CR, Punt AE. Model performance for the determination of appropriate harvest levels in the case of data-poor stocks. Fisheries Research. 2011;110(2):342–355. doi: 10.1016/j.fishres.2011.04.024

[26] Wetzel CR, Punt AE. Performance of a fisheries catch-at-age model (Stock Synthesis) in data-limited situations. Marine and Freshwater Research. 2011;62(8):927–936. doi: 10.1071/MF11006

[27] Hilborn R, Stewart IJ, Branch TA, Jensen OP. Defining Trade-Offs among Conservation, Profitability, and Food Security in the California Current Bottom-Trawl Fishery. Conservation Biology. 2012;26(2):257–268. doi: 10.1111/j.1523-1739.2011.01800.x 22443131 10.1111/j.1523-1739.2011.01800.x

[28] Melnychuk MC, Banobi JA, Hilborn R. Effects of Management Tactics on Meeting Conservation Objectives for Western North American Groundfish Fisheries. PLOS ONE. 2013;8(2):1–15. doi: 10.1371/journal.pone.0056684 10.1371/journal.pone.0056684 PMC3584066 23460809

[29] Carruthers TR, Punt AE, Walters CJ, MacCall A, McAllister MK, Dick EJ, et al Evaluating methods for setting catch limits in data-limited fisheries. Fisheries Research. 2014;153:48–68. doi: 10.1016/j.fishres.2013.12.014

[30] Thorson JT, Jensen OP, Hilborn R. Probability of stochastic depletion: an easily interpreted diagnostic for stock assessment modelling and fisheries management. ICES Journal of Marine Science: Journal du Conseil. 2015;72(2):428–435. doi: 10.1093/icesjms/fsu127

[31] Sethi SA, Branch TA, Watson R. Global fishery development patterns are driven by profit but not trophic level. Proceedings of the National Academy of Sciences. 2010;107(27):12163–12167. doi: 10.1073/pnas.1003236107 10.1073/pnas.1003236107 PMC2901455 20566867

[32] Dulvy NK, Fowler SL, Musick JA, Cavanagh RD, Kyne PM, Harrison LR, et al Extinction risk and conservation of the world’s sharks and rays. eLife. 2014;3:e00590 doi: 10.7554/eLife.00590 24448405 10.7554/eLife.00590 PMC3897121

[33] Cortes E. Stock assessment of small coastal sharks in the U.S. Atlantic and Gulf of Mexico. National Marine Fisheries Service, Southeast Fisheries Science Center; 2002. SFD-01/02-152. Available from: http://www.nmfs.noaa.gov/sfa/hms/species/sharks/documents/scs_assessment_rev2.pdf

[34] Clark WG. The effect of recruitment variability on the choice of a target level of spawning biomass per recruit In: Kruse G, Engers DM, Marasco RJ, Pautzke C, Quinn TJI, editors. Proceedings of the International Symposium on Management Strategies for Exploited Fish Populations. Fairbanks, AK: University of Alaska, Alaska Sea Grant Report 93-02; 1993 p. 233–246.

[35] Conway F, Shaw W. Socioeconomic Lessons Learned from the Response to the Federally-Declared West Coast Groundfish Disaster. Fisheries. 2008;33(6):269–277. doi: 10.1577/1548-8446-33.6.269

[36] Doubleday ZA, Prowse TAA, Arkhipkin A, Pierce GJ, Semmens J, Steer M, et al Global proliferation of cephalopods. Current Biology. 2016;26(10):R406–R407. doi: 10.1016/j.cub.2016.04.002 27218844 10.1016/j.cub.2016.04.002

[37] Rosenbaum PR, Rubin DB. The central role of the propensity score in observational studies for causal effects. Biometrika. 1983;70(1):41–55. doi: 10.1093/biomet/70.1.41

[38] Melnychuk MC, Essington TE, Branch TA, Heppell SS, Jensen OP, Link JS, et al Can catch share fisheries better track management targets? Fish and Fisheries. 2012;13(3):267–290. doi: 10.1111/j.1467-2979.2011.00429.x

[39] Cook RM, Heath MR. Population trends of bycatch species reflect improving status of target species. Fish and Fisheries. 2018 doi: 10.1111/faf.12265

[40] Kokkalis A, Eikeset AM, Thygesen UH, Steingrund P, Andersen KH. Estimating uncertainty of data limited stock assessments. ICES Journal of Marine Science. 2017;74(1):69–77.

