# Supplementary material for: Drivers and rates of stock assessments in the United States
Source: PLoS One. 2018 May 11;13(5):e0196483. doi: 10.1371/journal.pone.0196483 (PMC5947900; doi:10.1371/journal.pone.0196483)
Supplement: S1 Table — (PDF) [file pone.0196483.s002.pdf]

S1 Table: Paramter estimates

Philipp Neubauer[1*], James T. Thorson[2], Michael C. Melnychuk[3], Richard Methot[2], Kristan Blackhart[4]

1 Dragonfly Data Science, Wellington, New Zealand
2 NOAA Northwest Fisheries Science Center, Seattle, WA, USA
3 School of Aquatic and Fisheries Science, University of Washington, Seattle, WA, USA
4 ECS Federal, INC., Fairfax, VA, USA, on behalf of NOAA Fisheries, Office of Science and Technology

* philipp@dragonfly.co.nz

Posterior means of model parameters under interpretations of ratio of rates (θ) or time-to-assessment (ν), and probability $P(\theta > 1)$ that increasing parameter values or stocks in a given category have an increased likelihood of assessment compared to the baseline. Under the ratio of rates interpretation, the rate effect θ represents rates at which stocks with different characteristics are assessed relative to a baseline of 1. Under the time-to-assessment interpretation, the time effect ν is a multiplicative acceleration factor, i.e., $\nu = 0.5$ suggests a stock with these characteristics is assessed twice as fast as the average stock.

Effect	Category	Rate effect (θ)	Time effect (ν)	$P(\theta > 1)$
Region	Southeast	1.25	0.92	0.77
Region	Alaska	1.20	0.93	0.73
Region	Northeast	1.01	1.00	0.51
Region	West Coast	0.64	1.18	0.08
Habitat	Benthic	3.68	0.61	0.99
Habitat	Reef	1.03	0.99	0.53
Habitat	Bathy-	0.94	1.02	0.44
Habitat	Pelagic	0.74	1.12	0.25
Habitat	Benthopelagic	0.65	1.18	0.15
Habitat	Demersal	0.58	1.23	0.08
Class	Actinopterygii	0.76	1.11	0.35
Class	Bivalvia	0.25	1.71	0.08
Class	Branchiopoda	0.76	1.11	0.40
Class	Cephalaspidomorphi	0.81	1.08	0.43
Class	Cephalopoda	4.27	0.57	0.88
Class	Echinoidea	2.21	0.74	0.77
Class	Elasmobranchii	1.29	0.91	0.62
Class	Gastropoda	0.94	1.02	0.47
Class	Holocephali	0.42	1.40	0.26
Class	Holothuroidea	11.32	0.39	0.90
Class	Malacostraca	0.76	1.11	0.38
Class	Merostomata	0.48	1.32	0.26

Continued on next page

Table 1 – continued from previous page

Effect	Category	Rate effect (θ)	Time effect (ν)	$P(\theta > 1)$
Order	Acipenseriformes	0.77	1.11	0.40
Order	Anguilliformes	0.40	1.42	0.13
Order	Anostraca	0.85	1.07	0.44
Order	Arcoida	0.93	1.03	0.47
Order	Aspidochirotida	2.57	0.70	0.80
Order	Beloniformes	0.55	1.26	0.23
Order	Carcharhiniformes	3.56	0.62	0.97
Order	Chimaeriformes	0.68	1.16	0.35
Order	Clupeiformes	0.92	1.03	0.43
Order	Cypriniformes	1.02	0.99	0.50
Order	Cyprinodontiformes	0.83	1.07	0.42
Order	Decapoda	0.91	1.04	0.45
Order	Echinoida	1.55	0.85	0.67
Order	Elopiformes	0.42	1.40	0.13
Order	Gadiformes	2.37	0.72	0.97
Order	Lamniformes	0.87	1.05	0.44
Order	Lampriformes	0.85	1.06	0.44
Order	Lophiiformes	1.64	0.83	0.74
Order	Mugiliformes	0.55	1.26	0.17
Order	Myliobatiformes	0.97	1.01	0.49
Order	Myoida	1.74	0.81	0.75
Order	Mytiloida	0.65	1.18	0.32
Order	Neogastropoda	0.95	1.02	0.48
Order	Ophidiiformes	0.83	1.07	0.43
Order	Orectolobiformes	0.98	1.01	0.49
Order	Osmeriformes	0.61	1.20	0.29
Order	Ostreoida	0.46	1.34	0.18
Order	Perciformes	1.08	0.97	0.57
Order	Petromyzontiformes	0.94	1.03	0.48
Order	Pleuronectiformes	4.05	0.59	1.00
Order	Pristiformes	0.95	1.02	0.47
Order	Rajiformes	0.90	1.04	0.45
Order	Scorpaeniformes	6.06	0.50	1.00
Order	Squaliformes	0.73	1.12	0.34
Order	Squatiniformes	0.77	1.10	0.39
Order	Tetraodontiformes	0.90	1.04	0.45
Order	Teuthida	2.13	0.75	0.76
Order	Veneroida	0.86	1.06	0.42
Order	Xiphosura	0.70	1.14	0.36
Order	Zeiformes	0.90	1.04	0.46
Fixed effect	Mean ex-vessel price	5.07	0.54	1.00
Fixed effect	Maximum landings	4.14	0.58	1.00
Fixed effect	Maximum length	1.86	0.79	0.99
Fixed effect	Landings x Price	0.19	1.89	0.00
