# Supplementary material for: Drivers and rates of stock assessments in the United States
Source: PLoS One. 2018 May 11;13(5):e0196483. doi: 10.1371/journal.pone.0196483 (PMC5947900; doi:10.1371/journal.pone.0196483)
Supplement: S1 Appendix — (PDF) [file pone.0196483.s003.pdf]

# S1 Appendix: Assignment of year of first stock assessment

Philipp Neubauer[1*], James T. Thorson[2], Michael C. Melnychuk[3], Richard Methot[2], Kristan Blackhart[4]

**1** Dragonfly Data Science, Wellington, New Zealand
**2** NOAA Northwest Fisheries Science Center, Seattle, WA, USA
**3** School of Aquatic and Fisheries Science, University of Washington, Seattle, WA, USA
**4** ECS Federal, INC., Fairfax, VA, USA, on behalf of NOAA Fisheries, Office of Science and Technology

* philipp@dragonfly.co.nz

We used a three-step process to classify stocks as assessed or unassessed and, for assessed stocks, to identify the year of first assessment.

1. Management attribute database and interviews: As part of a larger data collection effort to characterize management attributes at the stock level, we conducted a survey which included the question "Year of first stock assessment", with a description of what qualifies as an assessment (as outlined in the main text). To answer this question, we first looked through historical stock assessments archived on fisheries agency websites to identify the first assessment that met our defined criteria (as not all published assessments would meet our relatively strict criteria). Websites accessed included those of US Fisheries Management Councils, NMFS Science Centers, and state-level fisheries management agencies. To confirm our findings, or if we were unable to answer the question from archived assessments, we conducted interviews with fisheries scientists and managers familiar with one or more stocks in each region. Individuals who participated in interviews for management attributes for US stocks are listed in our Acknowledgments. These surveys were conducted for 196 US stocks, of which 165 were found to have a stock assessment as defined. The intention of these surveys was to cover primarily well-studied stocks and stocks important to regional fisheries, not to draw a random sample from the NOAA landings data. Therefore, the proportion of these stocks meeting the criteria of having a stock assessment is high compared to the proportion of stocks from the NOAA landings database that are assessed.

2. Comparison with SIS database: We compared our list of assessed stocks with the list of assessed stocks from the US Species Information System (SIS) database. We considered SIS stocks with assessment level 3 or greater (see **??**). We found 31 stocks in the SIS database that were not previously included in our list of assessed stocks in (1). Most of these stocks were first assessed after our period of data collection in (1), 2012–2015, which is why many of these were not originally included in our list of stocks in (1). We added these 31 stocks along with their year of first assessment to our dataset.

3. Remaining stocks in NOAA landings database: To ensure that we had not overlooked any US marine stocks with a stock assessment (including stocks managed by state agencies), we systematically searched online for a stock

assessment for each species in the NOAA landings database. These searches comprised two types of species: some species in the NOAA landings database were not previously included in our collection of stock management attribute data in (1) or (2); and some species in the NOAA landings database were previously included in (1) or (2) but also had landings recorded in states outside of the defined areas of distribution of those stocks, and thus other assessments of the same species may have been available, for other areas. For both types of species, we searched for any stock assessment corresponding to species $\times$ state recorded landings that were unaccounted for in (1) or (2). Online searches consisted of: (i) going through assessment archives on the websites of US Fisheries Management Councils, NMFS Science Centers, and state-level fisheries management agencies; and (ii) using the Google search engine with search terms ("Latin name of species" "stock assessment" "Region") to identify available assessments either in the primary literature or that may not have been otherwise available through agency websites. In addition to confirming many of the added assessments in (2), we found 15 stock assessments meeting our criteria for assessment which were not previously accounted for. Many of these were stocks managed by state agencies so did not appear in the list in (2). These 15 stocks were added to our dataset along with their year of first assessment.

In total, after steps 1–3 we generated a list of 242 US stocks, including 211 stocks with stock assessments that qualified under our defined criteria. The remaining 31 stocks did not meet our assessment criteria. They were excluded from our dataset, although after merging our dataset of assessed stocks with the NOAA landings database, those stocks would later re-enter as unassessed stocks.
