# Supplementary material for: Drivers and rates of stock assessments in the United States
Source: PLoS One. 2018 May 11;13(5):e0196483. doi: 10.1371/journal.pone.0196483 (PMC5947900; doi:10.1371/journal.pone.0196483)
Supplement: S2 Appendix — (PDF) [file pone.0196483.s004.pdf]

# S2 Appendix: Validation of assessment classifications

Philipp Neubauer[1*], James T. Thorson[2], Michael C. Melnychuk[3], Richard Methot[2], Kristan Blackhart[4]

**1** Dragonfly Data Science, Wellington, New Zealand
**2** NOAA Northwest Fisheries Science Center, Seattle, WA, USA
**3** School of Aquatic and Fisheries Science, University of Washington, Seattle, WA, USA
**4** ECS Federal, INC., Fairfax, VA, USA, on behalf of NOAA Fisheries, Office of Science and Technology

* philipp@dragonfly.co.nz

In this appendix, we compare our assessment classifications with those of NOAA's Species Information System (SIS) database.

Our classification system consisted of whether or not a stock has had an assessment conducted, and subsequently, the year in which the first assessment occurred. Our definition of a stock assessment required the use of a population dynamics model fit to fishery landings data, coupled with some benchmark with which to compare model-estimated time series of abundance or fishing mortality (described further in the main text). These biological reference point benchmarks may have been estimated within the same assessment model or specified externally, and allow for comparing estimates of current abundance or fishing mortality relative to target levels. To determine the year of first stock assessment, we reviewed historical assessments on the websites of US Fishery Management Councils and NMFS Science Centers, and also sought the input of fishery scientists and managers within each region.

The SIS database uses a 6-level categorization of assessments [1], as follows:

0. Although some data may have been collected on this species, these data have not been examined beyond simple time series plots or tabulations of catch.
1. Either:
   a time series of a (potentially imprecise) abundance index calculated as raw or standardized CPUE in commercial, recreational, or survey vessel data, or
   b onetime estimation of absolute abundance made on the basis of tagging results, a depletion study, or some form of calibrated survey.
2. Simple equilibrium models applied to life history information; for example, yield per recruit or spawner per recruit functions based on mortality, growth, and maturity schedules; catch curve analysis; survival analysis; or length-based cohort analysis.
3. Equilibrium and non-equilibrium production models aggregated both spatially and over age and size; for example, the Schaefer model and the Pella-Tomlinson model.
4. Size, stage, or age structured models such as cohort analysis and untuned and tuned VPA analyses, age-structured production models, CAGEAN, stock synthesis, size or age-structured Bayesian models, modified DeLury methods, and size or age-based mark-recapture models.

5. Assessment models incorporating ecosystem considerations and spatial and seasonal analyses in addition to Levels 3 or 4. Ecosystem considerations include one or more of the following:

   a one or more time-varying parameters, either estimated as constrained series, or driven by environmental variables,

   b multiple target species as state variables in the model, or

   c living components of the ecosystem other than the target species included as state variables in the model."

We expect our classification of "assessed" to align with levels 3–5 in the SIS database, and our classification of "unassessed" to align with levels 0–2. This is generally what we found, and the discrepancies are described below. These comparisons can be followed in our final dataset provided (supplementary dataset), which list for each stock in our dataset the corresponding stock from the SIS database. In summary, the comparison shows:

I) Of the 211 stocks in our final dataset with a year of first stock assessment assignment, 189 have a corresponding stock in the SIS database. Of these 189 overlapping stocks, 171 (90%) had an assessment level of 3 or greater assigned in the SIS database. The 18 stocks that we classified as assessed but that have a current assessment level less than 3 in the SIS database fall into three groups. First, the most recent assessment has been rejected during review for 7 stocks that would have otherwise had a SIS assessment level of 3 or greater:

1. Atlantic cod - Georges Bank
2. Hogfish - Carolinas
3. Blacknose shark - Gulf of Mexico
4. Blacktip shark - Atlantic
5. Bonnethead - Atlantic and Gulf of Mexico
6. Caribbean spiny lobster - Southern Atlantic Coast / Gulf of Mexico
7. Atlantic halibut - Northwestern Atlantic Coast

Because qualifying assessments for these stocks have been accepted and used for management in the past, we treat these as assessed stocks in our analysis and maintain their year of first assessment.

Second, 10 stocks currently have a SIS assessment level less than 3 despite having had a qualifying assessment in the past:

1. Silver hake - Gulf of Maine / Northern Georges Bank
2. Silver hake - Southern Georges Bank / Mid-Atlantic
3. Atlantic croaker - Mid-Atlantic Coast
4. Atlantic mackerel - Gulf of Maine / Cape Hatteras
5. Winter flounder - Gulf of Maine

8. Northern shortfin squid - Northwestern Atlantic Coast

9. Red deepsea crab - Northwestern Atlantic

10. Horseshoe crab - Atlantic

The reasons for these discrepancies can be attributed to a population model (e.g. VPA) coupled with biological reference points previously used in the stock assessment process to inform fisheries management, but currently assessments rely on simpler models with lower assessment level categories in the SIS database (e.g. index-based methods or simpler equilibrium models).

Third, one additional stock that was assessed in 2016 for the first time is not in a federal FMP and not included in SIS as having had an assessment: White seabass - Pacific Coast.

II) Of the 358 stocks in our final dataset that were considered unassessed (based on species in the NOAA landings-by-state database that did not pair with assessed stocks) 143 have a corresponding stock in the SIS database. Of these 143 overlapping stocks, 138 (97%) had an assessment level of 2 or less assigned in the SIS database, and an additional 3 (2%) had a recent assessment rejected that would have been a level 4 assessment had it not been rejected. The two stocks that we classified as unassessed but that have an assessment level of 3 or 4 in the SIS database are:

1. Yelloweye rockfish - Bering Sea / Aleutian Islands; Yelloweye rockfish - Gulf of Alaska

2. Stone crabs (Menippe spp.) - Gulf of Mexico

Stock assessments for these stocks are based on area-swept biomass estimates from surveys, other index-based methods, or do not appear to be coupled with biological reference points to meet our definition of a full assessment. To be consistent with other stocks in our analysis, we consider these stocks as unassessed.

# References


1. NOAA National Marine Fisheries Service. Species Information System Public Portal; 2015.
   `https://www.st.nmfs.noaa.gov/sisPortal/sisPortalMain.jsp`.
