# Supplementary figures and images for: Drivers and rates of stock assessments in the United States
Source: PLoS One. 2018 May 11;13(5):e0196483. doi: 10.1371/journal.pone.0196483 (PMC5947900; doi:10.1371/journal.pone.0196483)

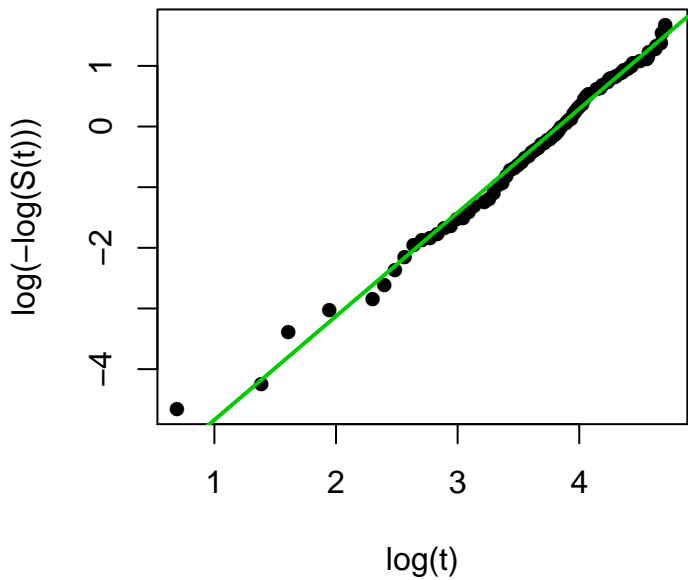

Supplement: S1 Fig — If the Weibull applies, the time from first landings (or from first quantitative stock assessment in 1960 if a stock was landed before 1960) to the year of first assessment should fall on a line with slope τ (the Weibull shape parameter) between log(-log(S^(t))), where S^(t) is the non-parametric Kaplan-Meyer estimate of survival at time t, and the log of t. Here, τ evaluates to 1.71 (slope of the green line), suggesting an increasing assessment rate with increasing time t. (PDF) [file pone.0196483.s005.pdf]

Cumulative hazard

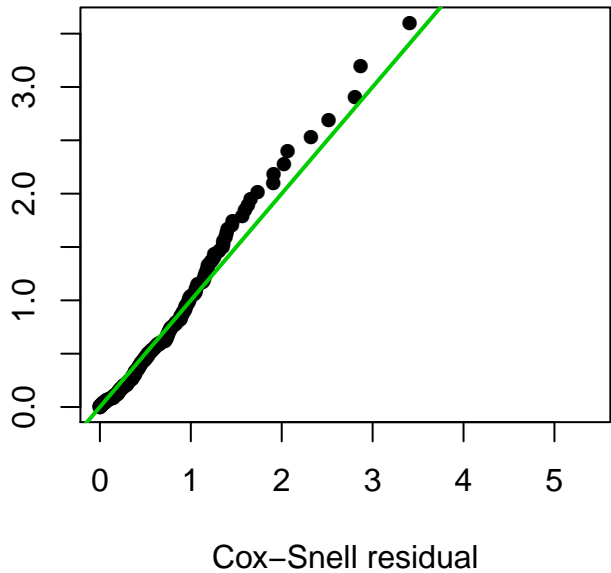

Supplement: S2 Fig — For a perfect fit all data points would lie on the y = x (green) line. (PDF) [file pone.0196483.s006.pdf]

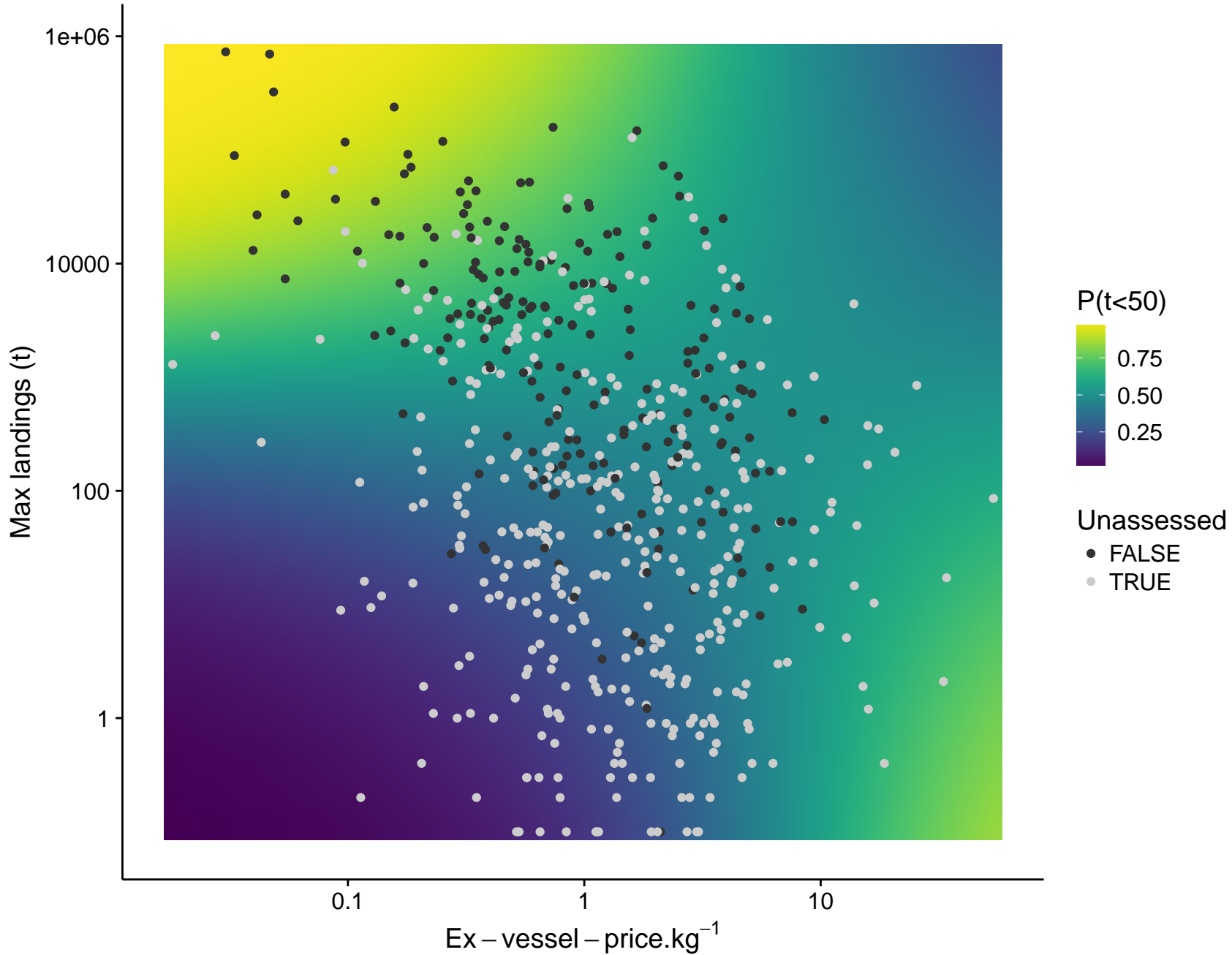

Supplement: S3 Fig — Marginal probabilities were evaluated at the mean of remaining continuous covariates. The dataset used for analysis is overlayed with assessed stocks as dark grey points and unassessed stocks in light grey. (PDF) [file pone.0196483.s007.pdf]

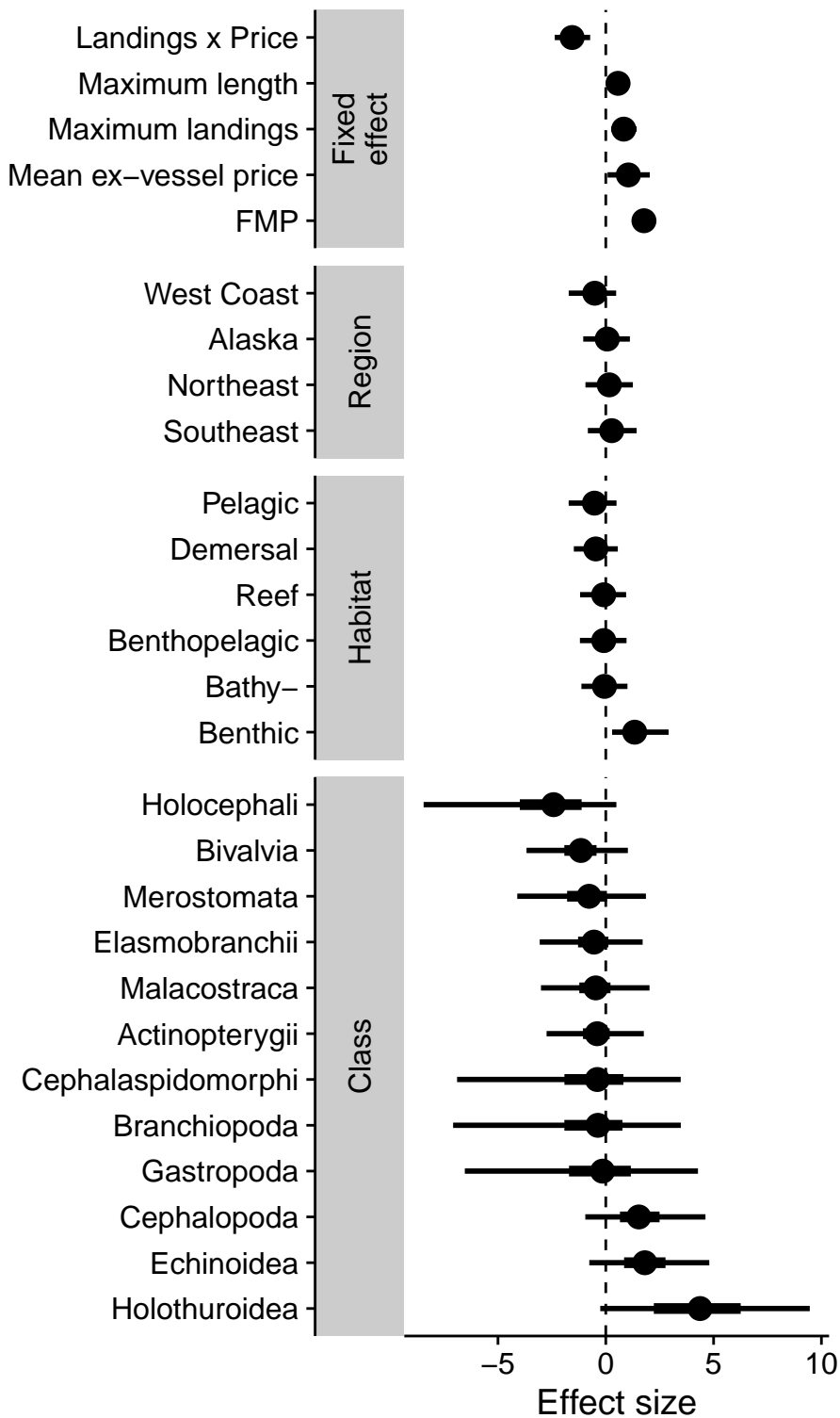

Supplement: S4 Fig — Summaries of estimated posterior distributions for fixed effects, regional random effects, habitat random effects, and taxonomic class random effects in the time-to-event model with fishery management plan (FMP) variable. Circles show posterior medians, thick bars show inter-quartile ranges of the posteriors, and thin lines show 95% confidence intervals. (PDF) [file pone.0196483.s008.pdf]

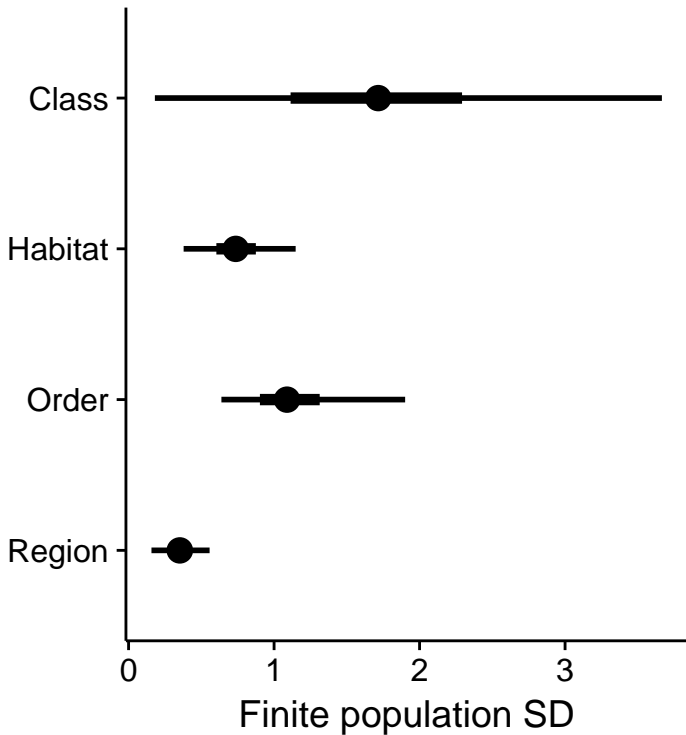

Supplement: S5 Fig — Circles show posterior medians, thick bars show inter-quartile ranges of the posteriors, and thin lines show 95% confidence intervals. (PDF) [file pone.0196483.s009.pdf]

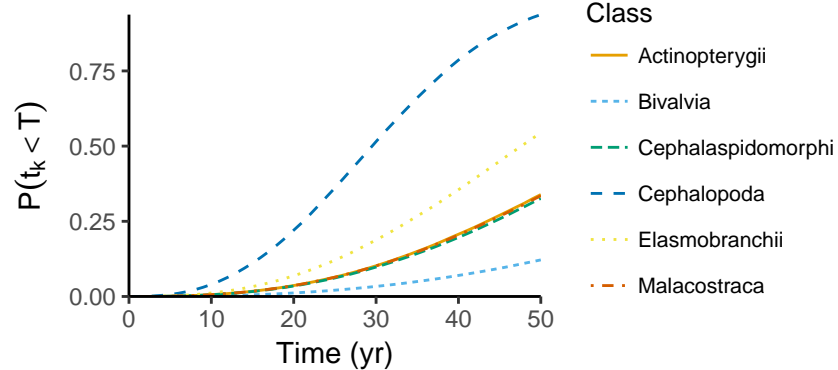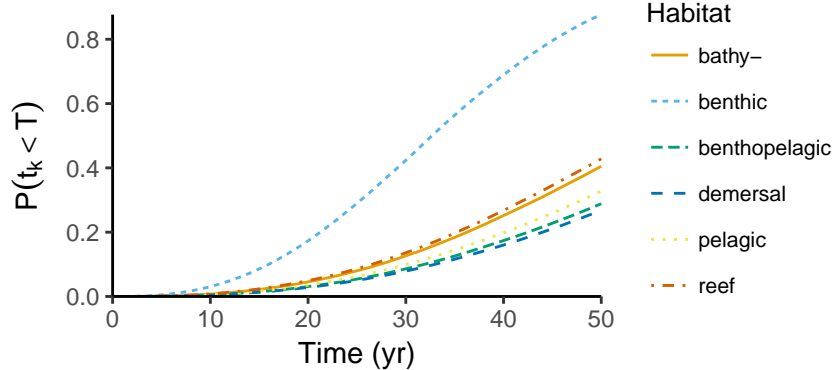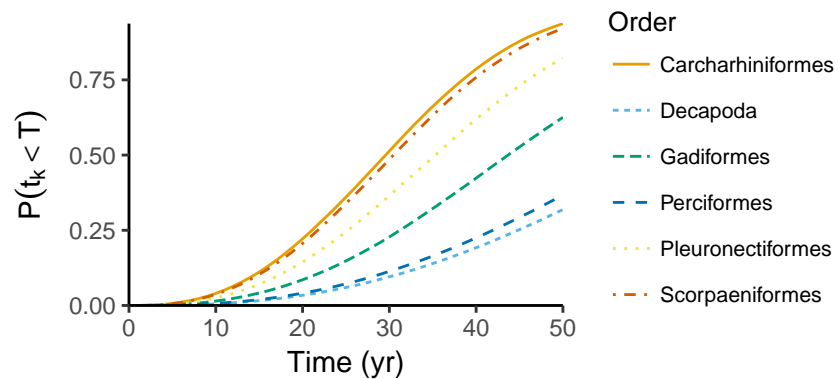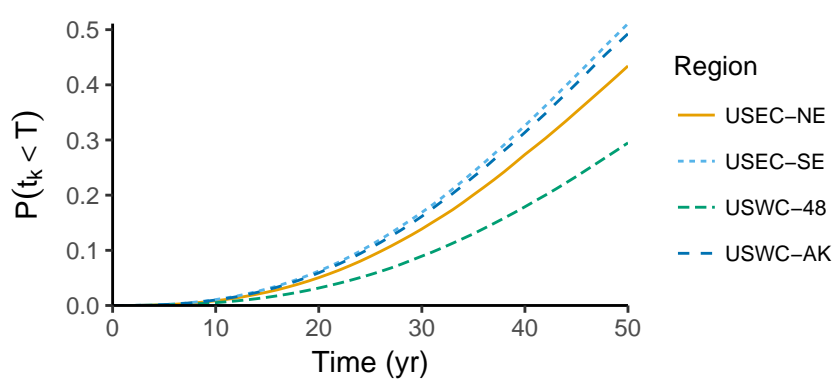

Supplement: S6 Fig — For taxonomic variables, only the six levels with the most stocks represented in our dataset are shown. Marginal probabilities were evaluated at the mean of (centered) continuous covariates. (PDF) [file pone.0196483.s010.pdf]
